# Efficacy and Safety of Evolocumab and Alirocumab as PCSK9 Inhibitors in Pediatric Patients with Familial Hypercholesterolemia: A Systematic Review and Meta-Analysis

**DOI:** 10.3390/medicina60101646

**Published:** 2024-10-08

**Authors:** Guoguang Xiao, Shan Gao, Yongmei Xie, Zhiling Wang, Min Shu

**Affiliations:** 1 Department of Pediatrics, West China Second Hospital, Sichuan University, Chengdu 610041, China; xggbcg@alu.scu.edu.cn (G.X.); huaxigaoshan@163.com (S.G.); may.xym@163.com (Y.X.); 2 Department of Pediatrics, West China Xiamen Hospital of Sichuan University, Xiamen 361022, China; 3 Key Laboratory of Birth Defects and Related Diseases of Women and Children (Sichuan University), Ministry of Education, Chengdu 610041, China

**Keywords:** familial hypercholesterolemia, evolocumab, alirocumab, child, efficacy, safety

## Abstract

*Background and Objectives*: The proprotein convertase subtilisin-kexin type 9 (PCSK9) inhibitors evolocumab and alirocumab are recently developed promising drugs used for treatment of familial hypercholesterolemia (FH). This systematic review and meta-analysis aimed to thoroughly evaluate the efficacy and safety of evolocumab and alirocumab among pediatric patients with FH. *Materials and Methods*: A comprehensive search was conducted in PubMed, Embase, CENTRAL (Cochrane Central Register of Controlled Trials), and ClinicalTrials.gov from inception through July 2024 to identify primary interventional studies among pediatric patients with FH. Meta-analyses were performed if appropriate. Statistics were analyzed using Review Manager version 5.4 and Stata version 16.0. *Results*: Fourteen articles reporting nine unique studies were included. There were three randomized controlled trials (RCTs) assessing evolocumab or alirocumab involving a total of 320 pediatric patients, one cross-over trial and five single-arm or observational studies. Pooled results showed significant efficacy of evolocumab/alirocumab in reducing low-density lipoprotein cholesterol (LDL-C) (weighted mean difference [WMD]: −37.92%, 95% confidence interval [CI]: −43.06% to −32.78%; I^2^ = 0.0%, *p* = 0.60), apolipoprotein B (WMD: −33.67%, 95% CI: −38.12% to −29.22%; I^2^ = 0.0%, *p* = 0.71), and also lipoprotein(a) (WMD: −16.94%, 95% CI: −26.20% to −7.69%; I^2^ = 0.0%, *p* = 0.71) among pediatric patients with FH. The efficacies of evolocumab/alirocumab on LDL-C reduction within pediatric patients with heterozygous FH (HeFH) were consistent between studies, whereas in patients with homozygous FH (HoFH), it varied dramatically. Pediatric patients with the null/null variant may respond to the treatment. PCSK9 inhibitors were generally well tolerated within most pediatric patients, in line with previous studies among adult populations. *Conclusions*: The PCSK9 inhibitors evolocumab/alirocumab significantly reduced LDL-C and some other lipid parameters, such as apolipoprotein B, in pediatric patients with HeFH. These drugs may be appropriate as a potential therapy for pediatric patients with HoFH who cannot achieve LDL-C targets with other treatments. Evolocumab/alirocumab was generally well tolerated in the pediatric population.

## 1. Introduction

Familial hypercholesterolemia (FH) is an autosomal dominant genetic disorder characterized by elevated plasma concentrations of low-density lipoprotein cholesterol (LDL-C) from birth and an increased risk of premature and progressive atherosclerotic cardiovascular disease (ASCVD) [1]. FH can be distinguished as heterozygous FH (HeFH) caused by the mutation of one allele of the FH gene, which is considered as the most common monogenic disorder, and the very rare but much more severe homozygous FH (HoFH) caused by two mutant alleles. Two recent meta-analyses including large populations have shown that the pooled prevalence of HeFH corresponds to one in around every 311–313 individuals, with an estimate of 6.8–8.5 million children and adolescents affected globally [2,3]. One baby with HeFH is born almost every minute worldwide [4]. In addition, HoFH occurs at a frequency of 1 in 250,000–360,000 cases [5].

FH is caused by mutations in genes encoding proteins involved in lipoprotein metabolism. About 90% of the patients with genetically diagnosed FH have mutations in the genes encoding the low-density lipoprotein receptor (LDLR). In addition, mutations in the genes encoding apolipoprotein B (APOB), proprotein convertase subtilisin-kexin type 9 (PCSK9), or low-density lipoprotein receptor adaptor protein 1 (LDLRAP1) have also been found to cause FH [1].

The raised level of LDL-C is the dominant factor determining to what extent the cardiovascular risk is increased. Patients with concentrations of LDL-C of more than 190 mg/dL) and no FH mutations had a six times higher risk of coronary artery disease compared with people with concentrations of LDL-C of less than 130 mg/dL and no mutations. And individuals with LDL-C concentrations of more than 190 mg/dL and genetically diagnosed FH had a 22 times increased risk [6].

Therefore, reducing LDL-C concentrations in childhood is considered to be of great importance for reducing cardiovascular risk [4,7]. Control of LDL-C concentrations among pediatric patients can prevent atherosclerosis progression and premature cardiovascular events in adulthood [7]. Furthermore, long-term lipid-lowering therapy (LLT) with statins reduces the progression of subclinical vascular diseases in children and adolescents [8]. Guidelines from the American College of Cardiology/American Heart Association define acceptable LDL-C concentrations in pediatric patients as less than 110 mg/dL [9]. Current pediatric guidelines from the European Atherosclerosis Society for management of FH recommend an LDL-C reduction to less than 130 mg/dL for patients over 10 years, or by at least 50% from baseline for patients from 8 years, especially among those with very high LDL-C and elevated lipoprotein(a) [4].

However, many pediatric patients with FH cannot achieve guideline-recommended LDL-C levels despite receiving high-dose statins and other LLTs such as ezetimibe. As a result, additional LLTs are needed for these child and adolescent patients to help control LDL-C concentrations and reduce the risk of ASCVD in adulthood.

PCSK9 is a protease that promotes the degradation of LDLR, which is responsible for the clearance of excess LDL-C from the blood, and also regulates the cell surface expression of lipid and lipoprotein receptors other than LDLR, impacting serum levels of multiple lipoprotein classes [10,11,12]. It has been reported that naturally occurring loss-of-function PCSK9 mutations are associated with low LDL-C levels from birth and a decreased risk of ASCVD in adulthood [13]. Inhibitors of PCSK9 are a recent development for the management of FH, and have been recommended to be used in very high-risk patients who fail to achieve their LDL-C targets while receiving maximally tolerated statin treatment with or without ezetimibe; when there is intolerance to the use of statins, PCSK9 inhibitors can be used as monotherapy or in combination with ezetimibe [5,14]. Two fully human monoclonal antibodies (mAbs) for inhibiting PCSK9, evolocumab and alirocumab, have been shown to reduce cardiovascular events in adult patients with ASCVD, and also LDL-C and other lipid parameters among pediatric patients with HeFH in randomized controlled trials (RCTs) [15,16,17,18]. Nevertheless, pediatric patients with HoFH showed remarkable variability in LDL-C reduction after treatment with these mAbs [19,20]. At present, studies about the impact of PCSK9 mAbs on the pediatric population with FH have been limited and often conducted in relatively small child and adolescent groups. The reported rates of reduction in lipid variables among these patients vary widely. More comprehensive assessments of the efficacy and safety of evolocumab and alirocumab in pediatric patients with FH are lacking.

Therefore, we conducted a systematic review, and meta-analysis if appropriate, to examine the cumulative evidence on the clinical efficacy and safety of the PCSK9 inhibitors evolocumab and alirocumab among pediatric patients diagnosed with FH to provide the basis for clinical practice.

## 2. Materials and Methods

This systematic review and meta-analysis was performed according to the established methods and standards recommended by the Cochrane Collaboration and the PRISMA (Preferred Reporting Items for Systematic Reviews and Meta-analyses) statement [21,22].

### 2.1. Data Sources and Searches

PubMed, Embase, CENTRAL (Cochrane Central Register of Controlled Trials), and ClinicalTrials.gov were comprehensively searched from inception through 30 July 2024 without language restriction. The search terms included the following, with the use of wildcard characters to account for variations in spelling and plurals: (PCSK9 inhibitor/antibody OR anti-PCSK9 OR evolocumab OR Repatha OR AMG145 OR alirocumab OR praluent OR SAR236553 OR REGN727) AND (familial hypercholesterolemia OR hypercholesterolemia type II) AND (child OR pediatric OR adolescent). Manual searches of reference lists and relevant review articles were conducted.

### 2.2. Study Selection

Two investigators (G.X. and S.G.) screened and selected the eligible studies independently, with disagreements resolved by discussion with a third investigator (Z.W.). Primary research studies investigating the impact, including efficacy and safety, of evolocumab/alirocumab on pediatric patients aged less than 18 years diagnosed with FH and providing aggregated outcomes were included. Studies conducted only within adult population and lacking sufficient information on the efficacy or safety for pediatric patients with FH, as well as duplicate publications, were excluded.

### 2.3. Data Extraction

Two investigators (G.X. and S.G.) independently extracted data by using a prespecified extraction form. Consensus was achieved through discussion with a third investigator (M.S.) in the case of discrepancies. The primary outcomes of interest were the reduction in LDL-C levels (from baseline to the end of treatment) and safety profiles of evolocumab/alirocumab among pediatric patients with FH. The data extracted were as follows: first author/trial name, year of publication, type of intervention, number of patients, mean age, disease type, treatment duration, patient characteristics, background LLT, endpoint including baseline and changes in lipid parameters and safety data, and funding information. The corresponding authors of the included studies were contacted for additional data if necessary.

### 2.4. Quality Assessment

Two authors (G.X. and Y.X.) independently assessed the quality of the included studies, and discrepancies were resolved through discussion with a third author (M.S.). The potential risk of bias of the included RCTs was assessed according to the Cochrane Collaboration guidelines. The following items including selection bias (randomization and allocation concealment), performance bias (blinding of participants and personnel), detection bias (blinding of outcome assessment), attrition bias (incomplete outcome data), and reporting bias (selective reporting) were assessed. The Newcastle–Ottawa scale was used for assessment of single-arm and observational studies [23]. No quality assessment was conducted for studies published in abstract form only.

### 2.5. Data Synthesis and Statistical Analysis

All analyses were performed using Review Manager version 5.4 (RevMan; Cochrane Collaboration) and Stata version 16.0 (Stata Corp, College Station, TX, USA). For the efficacy outcomes, the lipid parameters were considered as continuous variables and expressed as weighted mean difference (WMD) and 95% confidence interval (CI), and the results were pooled if appropriate. If not reported, standard deviation (SD) could be calculated from the CI, interquartile range (IQR), or standard error (SE) according to formulas proposed by the Cochrane Handbook. The odds ratio (OR) and 95% CI were used to statistically analyze the dichotomous data (safety outcomes). Heterogeneity was determined by the Cochran Q test and I2 statistic. I2 < 25% was considered as representing low heterogeneity, 25% < I2 < 75% representing moderate heterogeneity, and I2 > 75% representing high heterogeneity. The outcomes were analyzed by fixed-effects models under no or low inconsistency; otherwise, the data were pooled based on random-effects models. Nevertheless, both random- and fixed-effects models were computed as part of the sensitivity analysis. The sensitivity analysis was also performed by omitting studies in turn to evaluate the consistency of the pooled results. A *p* value < 0.05 was considered statistically significant. Due to the small number of eligible studies, we did not assess publication bias.

## 3. Results

### 3.1. Study Selection and Characteristics

**Figure 1:**
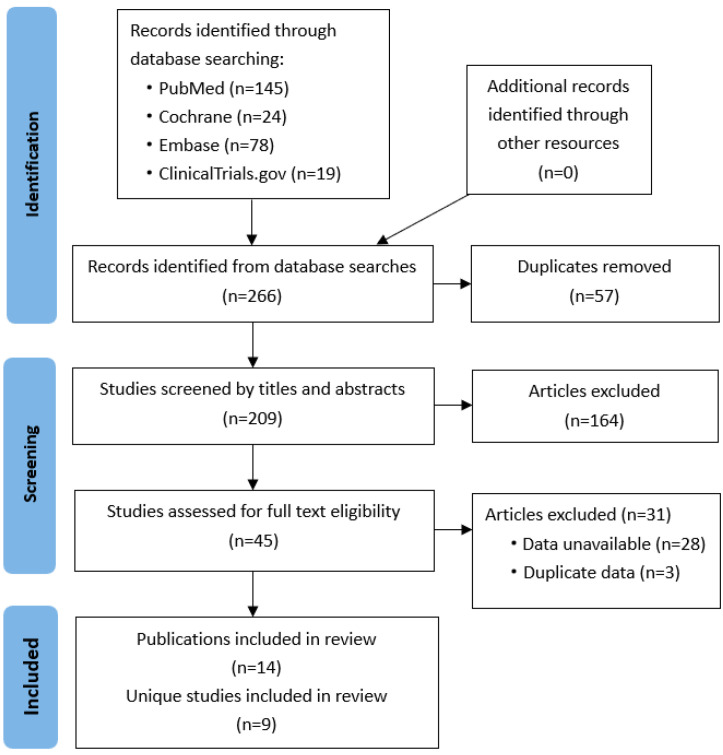
PRISMA flow diagram of study selection.

The initial literature search identified 266 possibly relevant publications. After excluding duplicate publications and screening the titles and abstracts, 45 articles were retrieved for full-text review. We further excluded 31 articles, of which 28 had no data on the efficacy and safety of PCSK9 inhibitors in pediatric patients with FH, and 3 were duplicate publications. At last, 14 articles reporting nine unique studies were included in the present study [17,18,19,20,24,25,26,27,28,29,30,31,32,33]. As shown in Table 1, some articles reported data of different aspects of the same study. The study identification process is shown in Figure 1.

A summary of the characteristics of the included studies is presented in Table 1. The publication date of these studies ranged from 2015 to 2024. There were four articles reporting the results of RCTs investigating the efficacy and safety of evolocumab/alirocumab among a total of 320 pediatric patients with FH, of whom 83% were white [17,18,24,25]. One study [24] investigating evolocumab in HoFH involved children and adults, and the corresponding author provided us with additional data about the adolescent participants. Another article reported two separate cohorts (each with an independent control group) that included patients receiving alirocumab every 2 weeks (Q2W) and every 4 weeks (Q4W), labelled as the Q2W cohort and Q4W cohort, respectively, which could be analyzed separately [18]. One randomized cross-over trial compared the efficacy of evolocumab and the newly developed PCSK9 inhibitor lerodacibep (LIB) among HoFH patients, including 19 adolescents, and was reported in abstract form only [28]. The remaining nine articles [19,20,26,27,29,30,31,32,33] reported five unique non-comparative studies involving relatively small numbers of participants except for the HAUSER-OLE study [31,32,33]. Most studies were conducted in multiple centers from different countries. Statins were the most commonly used background LLTs, with ezetimibe also being prescribed widely, as shown in Table 1. Overall, the patients’ adherence to prescribed medication regimens was high.

### 3.2. Study Quality and Risk-of-Bias Assessment

**Figure 2:**
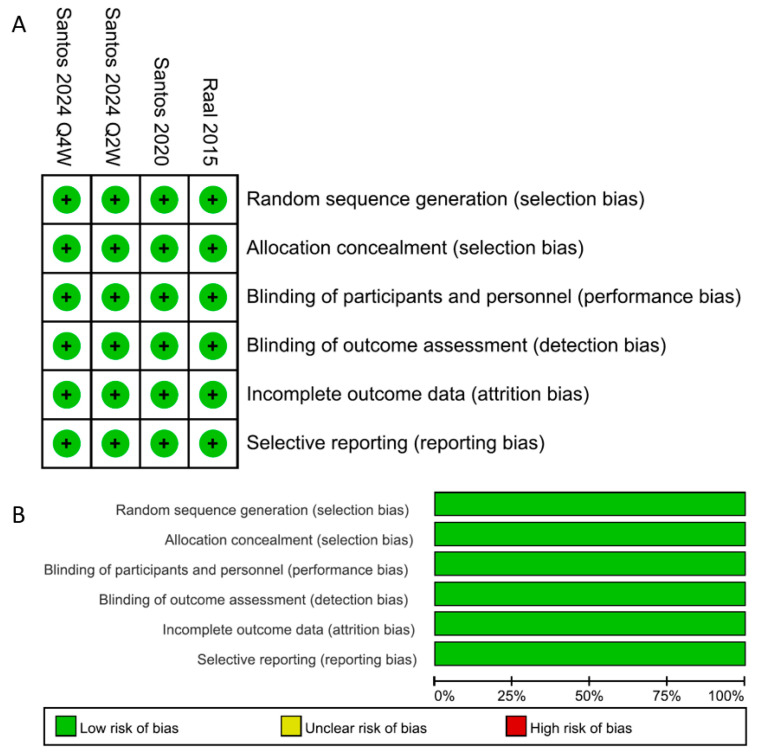
Risk-of-bias assessments of included RCTs. (**A**) 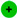 indicated low risk of bias. (**B**) Review authors’ judgements about each risk of bias item were presented as percentages across all included studies.

The three RCTs published in full text were assessed, using the Cochrane risk of bias tool for RCTs, as being of high methodological quality and low risk of bias for each assessed item, as shown in Figure 2. The non-comparative studies, assessed for quality using the Newcastle–Ottawa tool, were considered to have a relatively high risk of bias, as presented in Table 1.

### 3.3. Efficacy Outcomes

**Figure 3:**
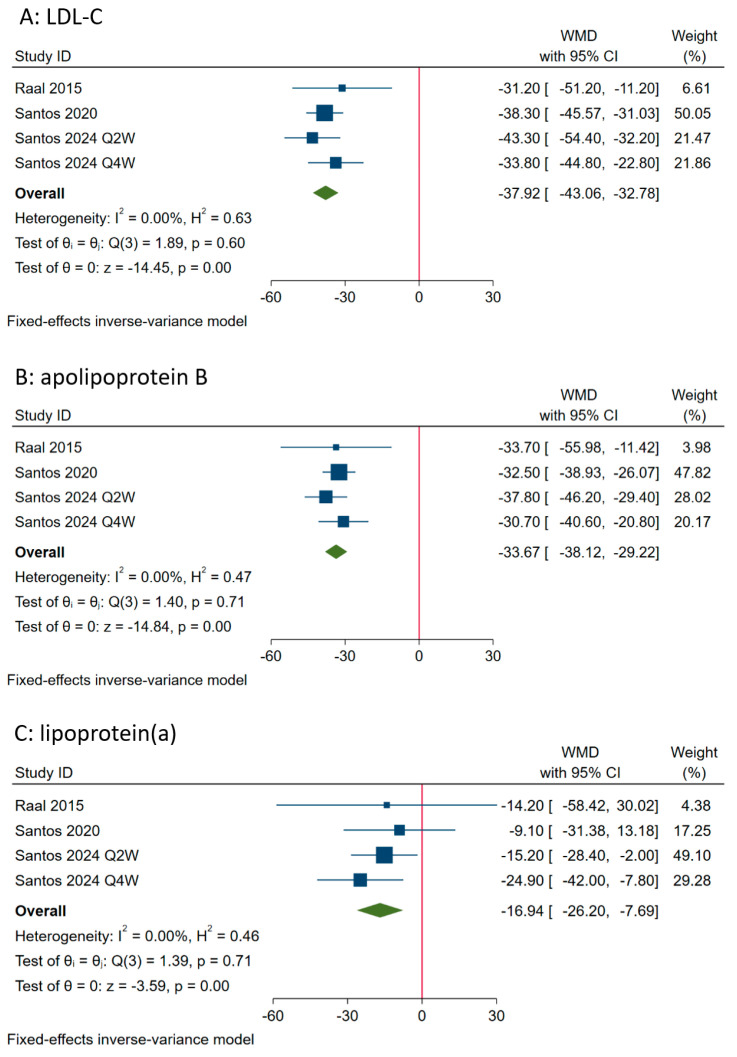
Forest plots showing the effect of evolocumab/alirocumab on percent reductions in lipid parameters. CI, confidence interval; LDL-C, low-density lipoprotein cholesterol; WMD, weighted mean difference [17,18,24].

Three RCTs [17,18,24] including four cohorts all reported the percentage changes in lipid parameters, including LDL-C, lipoprotein(a) and apolipoprotein B, from baseline to the end of treatment compared with placebo. One study [24] was conducted with HoFH patients and the other two [17,18] with HeFH patients. Evolocumab was investigated in two studies [17,24], and alirocumab in another RCT [18]. When the data were pooled with a fixed-effect model, evolocumab/alirocumab showed significant efficacy in reducing LDL-C (WMD: −37.92%, 95% CI: −43.06% to −32.78%; I^2^ = 0.0%, *p* = 0.60), apolipoprotein B (WMD: −33.67%, 95% CI: −38.12% to −29.22%; I^2^ = 0.0%, *p* = 0.71), and also lipoprotein(a) (WMD: −16.94%, 95% CI: −26.20% to −7.69%; I^2^ = 0.0%, *p* = 0.71), with low heterogeneity between each study, as shown in Figure 3. These results did not change markedly after using the random-effect model or omitting studies in turn. One dose-finding study assessing alirocumab in pediatric patients with HeFH demonstrated high reductions in LDL-C of −46% and −45% in the highest-dose cohorts, similar to reductions in apolipoprotein B of about −38% in these cohorts, and relatively small reductions in lipoprotein(a) of −14.5% or less [27]. The HAUSER-OLE study explored evolocumab in child patients with HeFH and showed a mean percent reduction in LDL-C of −35.3% and in apolipoprotein B of −25.1%, whereas the study found a mean percent increase in lipoprotein(a) of +16.8% from baseline to the end of the 80-week treatment [31]. After treatment, about 59.6–88.8% of pediatric patients with HeFH achieved an LDL-C level of less than 130 mg/dL [17,18,27,31]. The effects of evolocumab/alirocumab on other lipid parameters were also, but not consistently, reported in some studies, often with relatively small improvements.

**Figure 4:**
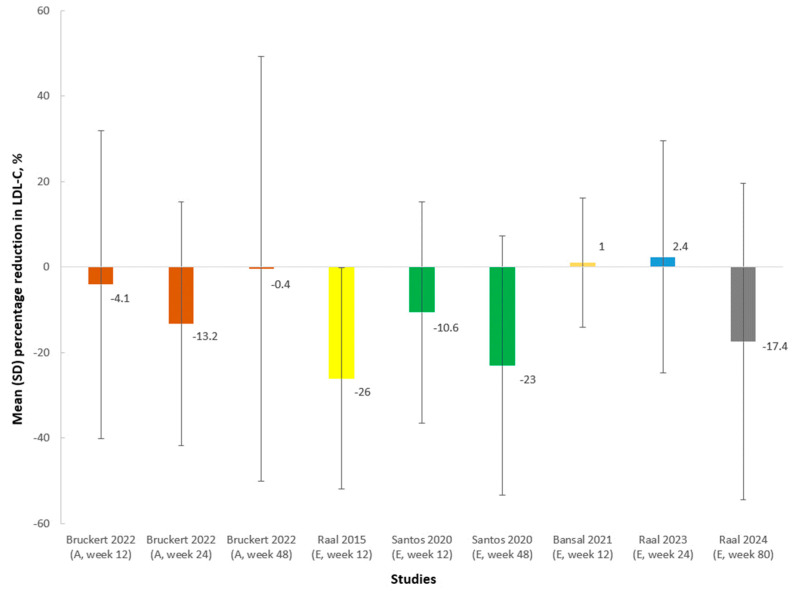
Mean (SD) percentage reduction in LDL-C in pediatric patients with HoFH compared with baseline (%) from different studies. Some studies reported mean (SD) percentage reduction in LDL-C at different time points (week 12, 24, or 48). The data are shown in the same color if from the same study. A, alirocumab; E, evolocumab; LDL-C, low-density lipoprotein cholesterol; SD, standard deviation [17,19,20,24,26,28].

**Figure 5:**
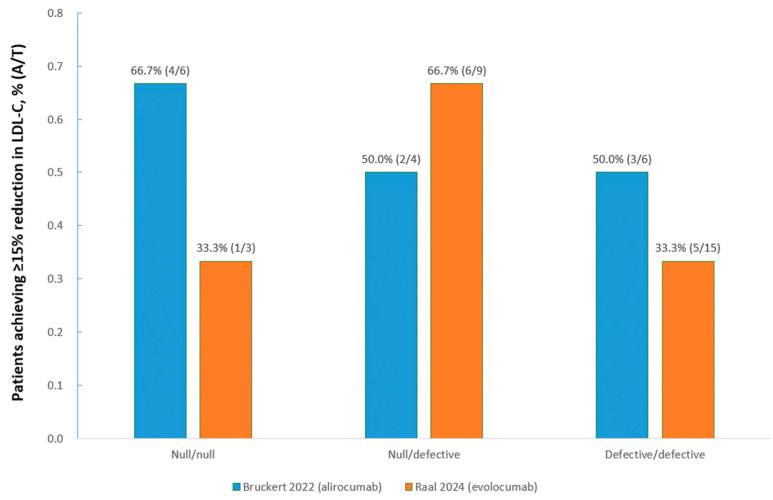
Pediatric HoFH patients with different LDLR gene types achieving ≥15% reduction in LDL-C. A, number of patients achieving ≥15% reduction in LDL-C for corresponding gene type; LDL-C, low-density lipoprotein cholesterol; T, total patients enrolled for corresponding gene type.

Six studies [10,20,24,26,28,29,30] investigated the efficacy of evolocumab/alirocumab on LDL-C in pediatric patients with HoFH, and the results varied greatly, with large SDs, indicating remarkable variability in response among this population, as illustrated in Figure 4. According to whether the LDLR activity in each allele was null or defective, HoFH were categorized as three types, namely defective/defective, defective/null, and null/null, and the effects of PCSK9 inhibitors on LDL-C could be related with residual LDLR activity [20]. One study [19] investigating alirocumab, and another report [20] pooling data from the TAUSSIG, RAMAN, and HAUSER-OLE studies assessing evolocumab, reported the numbers of pediatric patients with HoFH of different gene types who achieved at least a 15% reduction in LDL-C after treatment compared with baseline, respectively. A 15% reduction in LDL-C, translating into an absolute reduction of >60 mg/dL, is likely clinically meaningful [20]. The results showed that different kinds of treatment may lead to different efficacy among pediatric patients with different types of HoFH, as shown in Figure 5. There were relatively small efficacies of evolocumab/alirocumab on the reduction in other lipid variables, including apolipoprotein B and lipoprotein(a), in pediatric patients with HoFH, although the sample sizes were small [19,20].

Two studies [17,32] reported the effect of evolocumab on carotid intima-media thickness (cIMT) progression, an indicator of early ASCVD, in pediatric patients with HeFH. The results of HAUSER-RCT [17] showed that patients receiving evolocumab had a mean decrease of 0.003 mm (SD = 0.05) in cIMT summary score compared with a mean increase of 0.006 mm (SD = 0.05) in the placebo group from baseline to week 24, although without statistical significance (*p* = 0.403). However, in the HAUSER-OLE study, which was an open-label extension (OLE) study of HAUSER-RCT, all the participating patients received evolocumab, and the patients who had received a placebo during the RCT reversely achieved a mean reduction of 0.019 mm (SD = 0.04) in cIMT summary score after treatment from baseline to week 80, while the treatment group showed continued improvement during the OLE period [32].

### 3.4. Safety Data

**Figure 6:**
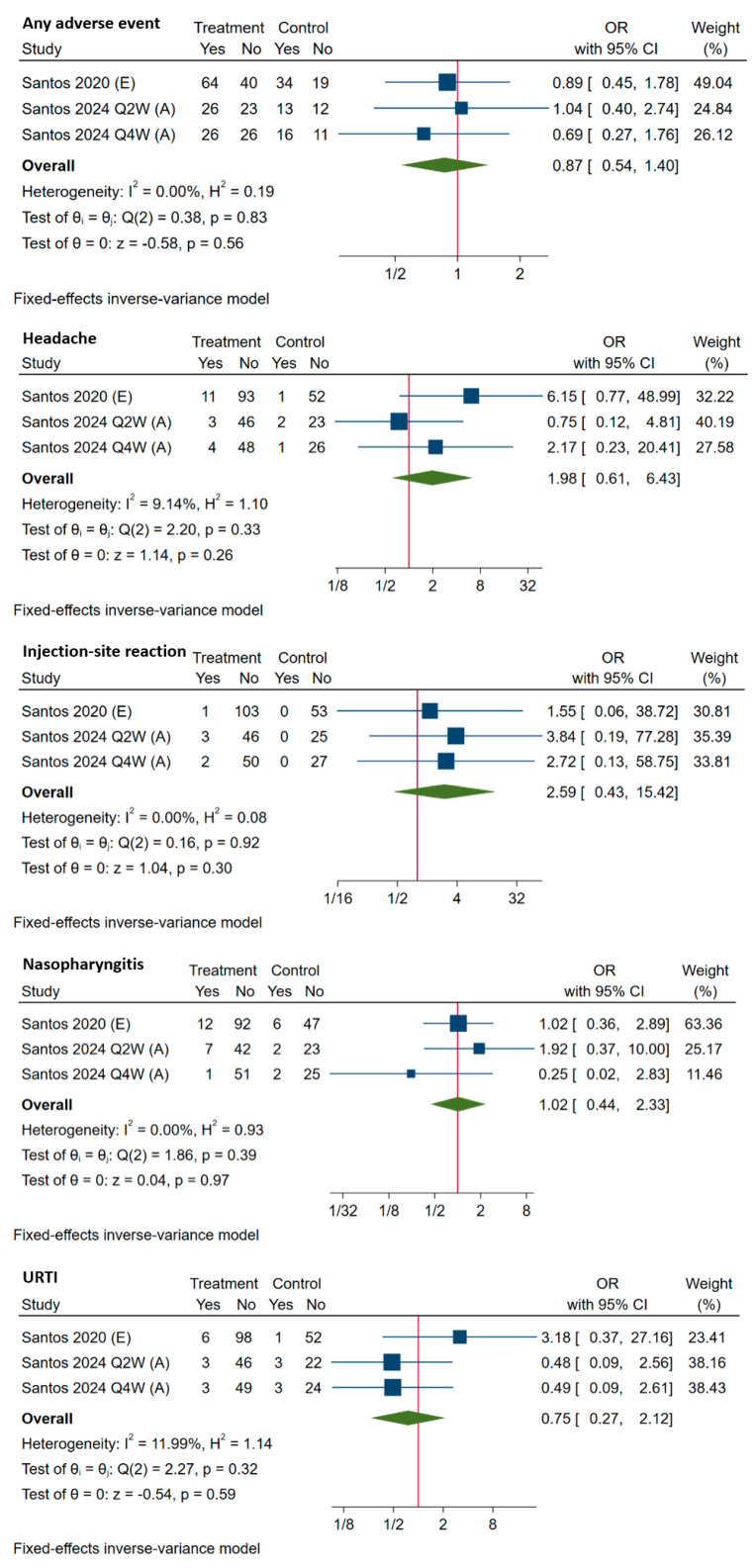
Comparisons of adverse events between evolocumab (E)/alirocumab (A) and placebo (control) groups. ‘Yes’ indicates the number of patients who presented with the adverse event; ‘No’ indicates the number of patients without the adverse event. CI, confidence interval; OR, odds ratio; URTI, upper respiratory tract infection [17,18].

Two of the included RCTs [17,18] involving three cohorts compared adverse events (AEs) between the evolocumab/alirocumab-treated group and a placebo group. The pooled results of commonly reported AEs, including any AE, headache, injection-site reaction, nasopharyngitis, and upper respiratory tract infection (URTI), showed no significant difference between the two groups, as shown in Figure 6. It should be noticed that no injection-site reactions were reported within the placebo group in these two RCTs, indicating that injection-site reaction could be related to the use of evolocumab/alirocumab, although at a low incidence and a nonserious level, as reported in other included studies [19,20,27,31].

No serious AEs were reported in two studies [24,27]. Nevertheless, syncope that was considered treatment related was reported in two pediatric cases receiving alirocumab, among whom one discontinued treatment [18]. Another study also reported one case of syncope among patients receiving evolocumab [20]. One study reported nonserious treatment-related arthropathy in one pediatric patient receiving evolocumab, leading to treatment discontinuation [17].

No changes with clinical importance were reported in the hematology and serum chemistry variables [17,27,31]. As to vitamin E, a decrease parallel to the decrease in LDL-C levels was observed in participants receiving alirocumab, but without vitamin E levels falling lower than the normal range [27]. However, in another comparative study, the levels of vitamin E were similar between the evolocumab and placebo groups [17]. No clinically important abnormalities were observed in electrocardiogram (ECG) measures among pediatric patients receiving evolocumab treatment [17,31].

No cases of diabetes were observed among patients receiving evolocumab in the HAUSER-RCT and HAUSER-OLE studies [17,31]. Daniels et al. [27] reported one case presenting with hyperglycemic events during the on-treatment period, and another patient was diagnosed with type 1 diabetes in the post-treatment period, both of which were considered by the investigators to be unrelated to the treatment with alirocumab.

The HAUSER-RCT and HAUSER-OLE studies reported that the treatment of evolocumab did not negatively influence cognitive functions [17,25,31]. But nonserious disturbances to attention and memory that were considered to be related to alirocumab treatment and that led to treatment discontinuation were reported in another study [18].

The growth parameters and Tanner stages of pubertal development were similar between the evolocumab/alirocumab and placebo groups, and remained appropriate for their age during the open-label follow-up studies [17,18,31]. However, another study noted a progression to a more advanced Tanner stage in a few pediatric patients receiving the alirocumab treatment [27].

Antibodies against evolocumab/alirocumab were not detected in most studies [17,18,24,31]. However, one study reported that positive antidrug antibodies were detected in 4 out of 42 pediatric patients receiving alirocumab during the treatment period, although not associated with safety concerns in these patients [27].

## 4. Discussion

After the first-line treatment with statins alone, many pediatric patients with FH remain unable to achieve the LDL-C goals because of limited drug response, side effects, or poor treatment adherence [34,35,36]. Additional LLTs can be given to pediatric patients who cannot attain the LDL-C targets with statins alone. Recently developed PCSK9 inhibitors including evolocumab and alirocumab may be considered as additional LLTs, as recommended by some guidelines [5,37]. Another type of PCSK9 mAb, bococizcumab, will not become available for clinical use because of a propensity of this drug for development of antidrug antibodies and a higher rate of injection-site reactions [38]. Inclisiran, a small interfering RNA against PCSK9, is being assessed among adolescents with FH by two ongoing studies [39]. Two important RCTs investigating evolocumab and alirocumab, respectively, among pediatric patients with HeFH, have been published recently [17,18]. The results showed significant efficacy of these two drugs for reducing LDL-C in these pediatric patients. However, studies on the efficacy and long-term safety of evolocumab/alirocumab in pediatric patients with FH are still limited and the results vary, especially among pediatric patients with HoFH.

The results of this systematic review and meta-analysis illustrate that the PCSK9 mAbs evolocumab/alirocumab could be efficacious in pediatric patients with HeFH in reducing LDL-C, in accordance with the findings among adult patients, although the LDL-C reductions in the pediatric patients seemed to be lower than those reported in adults [40,41]. The reasons for this difference in response to PCSK9 inhibitors between pediatric and adult patients diagnosed with HeFH are unclear. However, comparisons between clinical studies should be made cautiously in view of differences in study design and patient inclusion, particularly the differences in treatment targets and thresholds of examinations between pediatric and adult patients [18].

As shown in our analysis, evolocumab/alirocumab also led to significant reductions in apolipoprotein B, which is an established biomarker of cardiovascular risk [42], in line with another study involving adolescent and adult patients [43]. The pooled results of the RCTs in this analysis showed positive effects of evolocumab/alirocumab in reducing lipoprotein(a), which represents an independent risk factor for ASCVD in general [44]. These results showed low heterogeneity and were consistent with the results from other reports, mainly including adult patients, in which, however, high heterogeneities were detected [43,45]. Nevertheless, results from the single-arm HAUSER-OLE trial reported significant increases in lipoprotein(a) after treatment [31], and other studies with small sample sizes reported only small reductions in lipoprotein(a) after evolocumab/alirocumab treatment [19,20,27]. Therefore, the efficacy of these drugs on lipoprotein(a) in pediatric patients needs further research.

The pediatric patients diagnosed with HoFH responded poorly to PCSK9 mAbs compared with patients with HeFH. The target achievement of at least a 15% reduction in LDL-C, as recommended by the guidelines [5], was attained in nearly half of the pediatric patients with HoFH after treatment in two studies investigating evolocumab and alirocumab with percentage target achievements of 42.9% and 58.3%, respectively, among patients not receiving lipoprotein apheresis [19,23]. The results also demonstrated that LDL-C reduction after treatment with evolocumab/alirocumab in patients with HoFH was remarkably variable, with large SDs, and was difficult to predict. Some pediatric patients with the null/null mutation may respond to the treatment. The effects of evolocumab/alirocumab for individual patients could only be confirmed after treatment implementation, which might be due to the true genetic heterogeneity of the pediatric patients and differences in existing treatment [19]. It is recommended that PCSK9 inhibitors be considered in pediatric patients with HoFH but who are not responding adequately to high-intensity statin and ezetimibe therapy; if at least a 15% additional LDL-C reduction is achieved, PCSK9 inhibitor therapy may be continued, but if not, stopping this therapy should be considered [5].

Treatments with evolocumab/alirocumab were generally well tolerated in most pediatric patients, with a safety profile in accordance with that of previous studies among adult populations [29,40,46,47]. Injection-site reactions were reported in most included studies and all were nonserious with low incidence. Three cases of syncope were reported in the included studies, one of which led to treatment discontinuation. One study [27] reported a progression to a more advanced Tanner stage, decreased vitamin E levels comparable with the decrease in LDL-C, as well as positive antialirocumab antibodies in a few patients receiving alirocumab, although other studies did not make such findings. A previous study conducted in an adult population also reported low rates of immunogenicity of alirocumab and a sustained reduction in LDL-C levels regardless of antidrug-antibody status [48]. Nevertheless, these concerns should be monitored in future studies.

Furthermore, one case report described two 4-year-old patients with HoFH receiving evolocumab in a compassionate drug use program [49]. The two young patients achieved a percentage reduction in LDL-C of 30% or more and tolerated this treatment well. At present, evolocumab has not been approved for use in children younger than 10 years. If possible, studies evaluating evolocumab among these younger patients may be conducted in the future to benefit this population.

The present study had some limitations. Firstly, as both RCTs and noncomparative studies were included in this study, the data retrieved were heterogeneous, and interpretation of some individual studies was restricted due to methodological and reporting limitations. Secondly, despite the low heterogeneity detected in the meta-analysis, inherent methodological heterogeneity could be present owing to the pooling of results from different populations. Thirdly, it should be noted that all the included studies received fundings from drug companies, except one study [28] which did not report funding sources. In addition, more than 80% of the participants in this analysis were white, and due to national and ethnic differences, studies in other non-white child populations are needed. Finally, only a limited number of studies, many of which involved small sample sizes, were included in this study, and like other systematic reviews and meta-analyses, our study was a retrospective analysis. Therefore, prospective, large-scale, long-term, randomized trials are needed to further confirm these findings. The main strength of the present study was its systematic identification of eligible studies. Other strengths included standardized analyses, eligible studies being multicenter trials and the low heterogeneity identified between each study in the meta-analyses.

## 5. Conclusions

The present study demonstrated that the PCSK9 mAbs evolocumab/alirocumab significantly reduced LDL-C and some other lipid parameters such as apolipoprotein B in pediatric patients with HeFH. For pediatric patients with HoFH who cannot achieve LDL-C targets with other treatments, these drugs, as potential adjunct to LLTs, may be appropriate, even in those patients who would seem to gain limited benefit based on their LDLR functional status. Evolocumab/alirocumab was generally well tolerated in the pediatric population. In the future, large-scale, long-term RCTs of PCSK9 inhibitors are needed in different pediatric populations.

## Figures and Tables

**Table 1:** Characteristics of included studies.

Study Reference	Study Design	Intervention/Comparator	Treatment Duration	Mean Age (Years ± SD)	Disease Type	Background Treatment	Mean Baseline LDL-C ± SD (mg/dL)	Risk of Bias ^a^
Raal et al., 2015 [24] TESLA B NCT01588496	Multicenter, international, double-blind, placebo-controlled RCT	E: 420 mg Q4w (*n* = 7) PBO: Q4W (*n* = 3)	12 w	15.1 ± 1.4	HoFH	NA	E: 324.8 ± 132.7 PBO: 385.3 ± 166.6	See Figure 2
Santos et al., 2020 [17] HAUSER-RCT NCT02392559 Gaudet et al., 2022 [25]	Multicenter, international, double-blind, placebo-controlled RCT	E: 420 mg Q4w (*n* = 104) PBO: Q4W (*n* = 53)	24 w	13.7 ± 2.4	HeFH	Statin (*n* = 124) Ezetimibe (*n* = 21)	E: 185.0 ± 45.0 PBO: 183.0 ± 47.2	See Figure 2
Santos et al., 2024 [18] NCT03510884	Multicenter, international, double-blind placebo-controlled RCT	A: 40 mg (<50 kg) or 75 mg (≥50 kg) Q2w (*n* = 49) PBO: Q2W (*n* = 25)	24 w	12.8 ± 2.6	HeFH	Statin (*n* = 73) Ezetimibe (*n* = 5)	A: 169.7 ± 46.7 PBO: 175.3 ± 50.2	See Figure 2
		A: 150 mg (<50 kg) or 300 mg (≥50 kg) Q4W (*n* = 52) PBO: Q4W (*n* = 27)	24 w	13.0 ± 3.0	HeFH	Statin (*n* = 72) Ezetimibe (*n* = 16)	A: 176.8 ± 53.9 PBO: 176.6 ± 49.0	
Bansal et al., 2021 [26] RAMAN NCT03403374 Raal et al., 2024 [20]	Multicenter, single-country (India), open-label, single-arm trial	E: 420 mg Q4W (*n* = 13)	12 w	NA	HoFH	NA	NA	High (6)
Bruckert et al., 2022 [19] NCT03510715	Multicenter, international, open-label, single-arm trial	A: 75 mg (<50 kg) or 150 mg (≥50 kg) Q2W (*n* = 18)	48 w	12.4 ± 2.8	HoFH	Statin (*n* = 18) Ezetimibe (*n* = 15) Apheresis (*n* = 6)	373.0 ± 193.5	High (6)
Daniels et al., 2020 [27] ODYSSEY KIDS NCT02890992	Multicenter, international, open-label, dose-finding study	A: 30 mg (<50 kg) or 50 mg (≥50 kg) Q2W (*n* = 10)	8 w	12.7 ± 2.8	HeFH	Statin (*n* = 7) Ezetimibe (*n* = 4)	180.3 ±13.2	High (6)
		A: 40 mg (<50 kg) or 75 mg (≥50 kg) Q2W (*n* = 10)	8 w	13.1 ± 2.6	HeFH	Statin (*n* = 10)	160.0 ±12.0	
		A: 75 mg (<50 kg) or 150 mg (≥50 kg) Q2W (*n* = 11)	8 w	11.6 ± 2.7	HeFH	Statin (*n* = 11)	172.8 ±13.4	
		A: 150 mg (<50 kg) or 300 mg (≥50 kg) Q2W (*n* = 11)	12 w	12.4 ± 2.3	HeFH	Statin (*n* = 11)	188.9 ± 11.9	
Raal et al., 2023 [28] LIBerate-HoFH trial [Abstract]	International, randomized, cross-over trial	E: 420 mg Q4W (*n* = 19)	24 w	NA	HoFH	NA	482 ± 30.7	NA
Santos et al., 2020 [29] TAUSSIG NCT01624142 Raal et al., 2017 [30] [Abstract] Raal et al., 2024 [20]	Multicenter, international, open-label, single-arm trial	E: 420 mg Q4W (*n* = 10) or Q2W (*n* = 4) (Q2W for patients on apheresis)	260 w	NA	HoFH	Statin (*n* = 14) Ezetimibe (*n* = 13) Apheresis (*n* = 4)	351.9 ± 127.6	High (6)
Santos et al., 2022 [31] HAUSER-OLE NCT02624869 Wiegman et al., 2022 [32] [Abstract] Santos et al., 2024 [33] Raal et al., 2024 [20]	Multicenter, international, single-arm, open-label extension of HAUSER-RCT	E: 420 mg Q4W (*n* = 150)	80 w	14·0 ± 3.0	HeFH	Statin (*n* = 149) Ezetimibe (*n* = 21)	185.6 ± 46.4	High (6)
		E: 420 mg Q4W or Q2W (*n* = 12)	80 w	11.9 ± 1.7	HoFH	Statin (*n* = 12) Ezetimibe (*n* = 12)	406.8 ± 110.1	

A, alirocumab; E, evolocumab; HeFH, heterozygous familial hypercholesterolemia; HoFH, homozygous familial hypercholesterolemia; LDL-C, low-density lipoprotein cholesterol; NA, not available; PBO, placebo; Q2W, every 2 weeks; Q4W, every 4 weeks; RCT, randomized controlled trial; SD, standard deviation. ^a^ RCTs appraised using the Cochrane risk of bias tool for RCTs. Non-comparative trials appraised with Newcastle–Ottawa tool as being low (7–9), high (4–6), or very high (<4) risk of bias.

## Data Availability

All data generated or analyzed during this study are included in this article. Further enquiries can be directed to the corresponding author.

## References

[1] Defesche J.C., Gidding S.S., Harada-Shiba M., Hegele R.A., Santos R.D., Wierzbicki A.S. (2017). Familial hypercholesterolaemia. Nat. Rev. Dis. Primers.

[2] Beheshti S.O., Madsen C.M., Varbo A., Nordestgaard B.G. (2020). Worldwide Prevalence of Familial Hypercholesterolemia: Meta-Analyses of 11 Million Subjects. J. Am. Coll. Cardiol..

[3] Hu P., Dharmayat K.I., Stevens C.A.T., Sharabiani M.T.A., Jones R.S., Watts G.F., Genest J., Ray K.K., Vallejo-Vaz A.J. (2020). Prevalence of Familial Hypercholesterolemia among the General Population and Patients with Atherosclerotic Cardiovascular Disease: A Systematic Review and Meta-Analysis. Circulation.

[4] Wiegman A., Gidding S.S., Watts G.F., Chapman M.J., Ginsberg H.N., Cuchel M., Ose L., Averna M., Boileau C., Borén J. (2015). European Atherosclerosis Society Consensus Panel. Familial hypercholesterolaemia in children and adolescents: Gaining decades of life by optimizing detection and treatment. Eur. Heart J..

[5] Cuchel M., Raal F.J., Hegele R.A., Al-Rasadi K., Arca M., Averna M., Bruckert E., Freiberger T., Gaudet D., Harada-Shiba M. (2023). 2023 Update on European Atherosclerosis Society Consensus Statement on Homozygous Familial Hypercholesterolaemia: New treatments and clinical guidance. Eur. Heart J..

[6] Khera A.V., Won H.H., Peloso G.M., Lawson K.S., Bartz T.M., Deng X., van Leeuwen E.M., Natarajan P., Emdin C.A., Bick A.G. (2016). Diagnostic yield and clinical utility of sequencing familial hypercholesterolemia genes in patients with severe hypercholesterolemia. J. Am. Coll. Cardiol..

[7] Luirink I.K., Wiegman A., Kusters D.M., Hof M.H., Groothoff J.W., de Groot E., Kastelein J.J.P., Hutten B.A. (2019). 20-year follow-up of statins in children with familial hypercholesterolemia. N. Engl. J. Med..

[8] Rodenburg J., Vissers M.N., Wiegman A., van Trotsenburg A.S., van der Graaf A., de Groot E., Wijburg F.A., Kastelein J.J., Hutten B.A. (2007). Statin treatment in children with familial hypercholesterolemia: The younger, the better. Circulation.

[9] Grundy S.M., Stone N.J., Bailey A.L., Beam C., Birtcher K.K., Blumenthal R.S., Braun L.T., de Ferranti S., Faiella-Tommasino J., Forman D.E. (2019). 2018 AHA/ACC/AACVPR/AAPA/ABC/ACPM/ADA/AGS/APhA/ASPC/NLA/PCNA guideline on the management of blood cholesterol: Executive summary: A report of the American College of Cardiology/American Heart Association Task Force on Clinical Practice Guidelines. J. Am. Coll. Cardiol..

[10] Lagace T.A. (2014). PCSK9 and LDLR degradation: Regulatory mechanisms in circulation and in cells. Curr. Opin. Lipidol..

[11] Toth P.P., Hamon S.C., Jones S.R., Martin S.S., Joshi P.H., Kulkarni K.R., Banerjee P., Hanotin C., Roth E.M., McKenney J.M. (2016). Effect of alirocumab on specific lipoprotein non-high-density lipoprotein cholesterol and subfractions as measured by the vertical auto profile method: Analysis of 3 randomized trials versus placebo. Lipids Health Dis..

[12] Toth P.P., Sattar N., Blom D.J., Martin S.S., Jones S.R., Monsalvo M.L., Elliott M., Davis M., Somaratne R., Preiss D. (2018). Effect of evolocumab on lipoprotein particles. Am. J. Cardiol..

[13] Cohen J.C., Boerwinkle E., Mosley T.H., Hobbs H.H. (2006). Sequence variations in PCSK9, low LDL, and protection against coronary heart disease. N. Engl. J. Med..

[14] Banach M., Patti A.M., Giglio R.V., Cicero A.F.G., Atanasov A.G., Bajraktari G., Bruckert E., Descamps O., Djuric D.M., Ezhov M. (2018). International Lipid Expert Panel (ILEP). The role of nutraceuticals in statin intolerant patients. J. Am. Coll. Cardiol..

[15] Schwartz G.G., Steg P.G., Szarek M., Bhatt D.L., Bittner V.A., Diaz R., Edelberg J.M., Goodman S.G., Hanotin C., Harrington R.A. (2018). Alirocumab and cardiovascular outcomes after acute coronary syndrome. N. Engl. J. Med..

[16] Sabatine M.S., Giugliano R.P., Keech A.C., Honarpour N., Wiviott S.D., Murphy S.A., Kuder J.F., Wang H., Liu T., Wasserman S.M. (2017). Evolocumab and clinical outcomes in patients with cardiovascular disease. N. Engl. J. Med..

[17] Santos R.D., Ruzza A., Hovingh G.K., Wiegman A., Mach F., Kurtz C.E., Hamer A., Bridges I., Bartuli A., Bergeron J. (2020). Evolocumab in Pediatric Heterozygous Familial Hypercholesterolemia. N. Engl. J. Med..

[18] Santos R.D., Wiegman A., Caprio S., Cariou B., Averna M., Poulouin Y., Scemama M., Manvelian G., Garon G., Daniels S. (2024). Alirocumab in Pediatric Patients with Heterozygous Familial Hypercholesterolemia: A Randomized Clinical Trial. JAMA Pediatr..

[19] Bruckert E., Caprio S., Wiegman A., Charng M.J., Zárate-Morales C.A., Baccara-Dinet M.T., Manvelian G., Ourliac A., Scemama M., Daniels S.R. (2022). Efficacy and Safety of Alirocumab in Children and Adolescents with Homozygous Familial Hypercholesterolemia: Phase 3, Multinational Open-Label Study. Arterioscler. Thromb. Vasc. Biol..

[20] Raal F.J., Hegele R.A., Ruzza A., López J.A.G., Bhatia A.K., Wu J., Wang H., Gaudet D., Wiegman A., Wang J. (2024). Evolocumab Treatment in Pediatric Patients with Homozygous Familial Hypercholesterolemia: Pooled Data from Three Open-Label Studies. Arterioscler. Thromb. Vasc. Biol..

[21] Higgins J.P.T., Thomas J., Chandler J., Cumpston M., Li T., Page M.J., Welch V.A. (2023). Cochrane Handbook for Systematic Reviews of Interventions.

[22] Moher D., Liberati A., Tetzlaff J., Altman D.G., PRISMA Group (2009). Preferred reporting items for systematic reviews and meta-analyses: The PRISMA statement. Ann. Intern. Med..

[23] Stang A. (2010). Critical evaluation of the Newcastle-Ottawa scale for the assessment of the quality of nonrandomized studies in meta-analyses. Eur. J. Epidemiol..

[24] Raal F.J., Honarpour N., Blom D.J., Hovingh G.K., Xu F., Scott R., Wasserman S.M., Stein E.A., TESLA Investigators (2015). Inhibition of PCSK9 with evolocumab in homozygous familial hypercholesterolaemia (TESLA Part B): A randomised, double-blind, placebo-controlled trial. Lancet.

[25] Gaudet D., Ruzza A., Bridges I., Maruff P., Schembri A., Hamer A., Mach F., Bergeron J., Gaudet I., Pierre J.S. (2022). Cognitive function with evolocumab in pediatric heterozygous familial hypercholesterolemia. J. Clin. Lipidol..

[26] Bansal S., Ruzza A., Sawhney J., Kulkarni G., Iyengar S., Mehta V., Hamer A., Wu Y., Raal F.J. (2021). Evolocumab in patients with homozygous familial hypercholesterolemia in India. J. Clin. Lipidol..

[27] Daniels S., Caprio S., Chaudhari U., Manvelian G., Baccara-Dinet M.T., Brunet A., Scemama M., Loizeau V., Bruckert E. (2020). PCSK9 inhibition with alirocumab in pediatric patients with heterozygous familial hypercholesterolemia: The ODYSSEY KIDS study. J. Clin. Lipidol..

[28] Raal F.J., Mehta V., Kayikcioglu M., Blom D.J., Gupta P., Durst R., Asprusten E.A., Turner T., Bahassi E.M., Vest J. (2023). Response to PCSK9 Inhibition in Homozygous Familial Hypercholesterolemia Patients is Variable in Different Regions of the World and According to Age. Circulation.

[29] Santos R.D., Stein E.A., Hovingh G.K., Blom D.J., Soran H., Watts G.F., López J.A.G., Bray S., Kurtz C.E., Hamer A.W. (2020). Long-Term Evolocumab in Patients with Familial Hypercholesterolemia. J. Am. Coll. Cardiol..

[30] Raal F., Bruckert E., Blom D., Kurtz C., Coll B., Tang L., Somaratne R., Stein E.A. (2017). Evolocumab treatment in paediatric patients with homozygous familial hypercholesterolaemia: The Trial Assessing long-term Use of PCSK9 inhibition in Subjects with Genetic LDL disorders (TAUSSIG). Eur. Heart J..

[31] Santos R.D., Ruzza A., Hovingh G.K., Stefanutti C., Mach F., Descamps O.S., Bergeron J., Wang B., Bartuli A., Buonuomo P.S. (2022). Paediatric patients with heterozygous familial hypercholesterolaemia treated with evolocumab for 80 weeks (HAUSER-OLE): A single-arm, multicentre, open-label extension of HAUSER-RCT. Lancet Diabetes Endocrinol..

[32] Wiegman A., Ruzza A., Hovingh G.K., Santos R.D., Mach F., Stefanutti C., Luirink I., Bridges I., Wang B., Bhatia A.K. (2022). Evolocumab treatment reduces carotid intima-media thickness in paediatric patients with heterozygous familial hypercholesterolaemia. Eur. Respir. J..

[33] Santos R.D., Ruzza A., Wang B., Maruff P., Schembri A., Bhatia A.K., Mach F., Bergeron J., Gaudet I., St Pierre J. (2024). Evolocumab in paediatric heterozygous familial hypercholesterolaemia: Cognitive function during 80 weeks of open-label extension treatment. Eur. J. Prev. Cardiol..

[34] de Ferranti S.D., Shrader P., Linton M.F., Knowles J.W., Hudgins L.C., Benuck I., Kindt I., O’Brien E.C., Peterson A.L., Ahmad Z.S. (2021). Children with heterozygous familial hypercholesterolemia in the United States: Data from the Cascade Screening for Awareness and Detection-FH Registry. J. Pediatr..

[35] Ramaswami U., Futema M., Bogsrud M.P., Holven K.B., Roeters van Lennep J., Wiegman A., Descamps O.S., Vrablik M., Freiberger T., Dieplinger H. (2020). Comparison of the characteristics at diagnosis and treatment of children with heterozygous familial hypercholesterolaemia (FH) from eight European countries. Atherosclerosis.

[36] Bogsrud M.P., Langslet G., Wium C., Johansen D., Svilaas A., Holven K.B. (2018). Treatment goal attainment in children with familial hypercholesterolemia: A cohort study of 302 children in Norway. J. Clin. Lipidol..

[37] Horton A.E., Martin A.C., Srinivasan S., Justo R.N., Poplawski N.K., Sullivan D., Brett T., Chow C.K., Nicholls S.J., Pang J. (2022). Integrated guidance to enhance the care of children and adolescents with familial hypercholesterolaemia: Practical advice for the community clinician. J. Paediatr. Child Health.

[38] Ridker P.M., Revkin J., Amarenco P., Brunell R., Curto M., Civeira F., Flather M., Glynn R.J., Gregoire J., Jukema J.W. (2017). Cardiovascular efficacy and safety of bococizumab in high-risk patients. N. Engl. J. Med..

[39] Reijman M.D., Schweizer A., Peterson A.L.H., Bruckert E., Stratz C., Defesche J.C., Hegele R.A., Wiegman A. (2022). Rationale and design of two trials assessing the efficacy, safety, and tolerability of inclisiran in adolescents with homozygous and heterozygous familial hypercholesterolaemia. Eur. J. Prev. Cardiol..

[40] Kastelein J.J., Ginsberg H.N., Langslet G., Hovingh G.K., Ceska R., Dufour R., Blom D., Civeira F., Krempf M., Lorenzato C. (2015). ODYSSEY FH I and FH II: 78 week results with alirocumab treatment in 735 patients with heterozygous familial hypercholesterolaemia. Eur. Heart J..

[41] Raal F.J., Stein E.A., Dufour R., Turner T., Civeira F., Burgess L., Langslet G., Scott R., Olsson A.G., Sullivan D. (2015). PCSK9 inhibition with evolocumab (AMG 145) in heterozygous familial hypercholesterolaemia (RUTHERFORD-2): A randomised, double-blind, placebo-controlled trial. Lancet.

[42] Glavinovic T., Thanassoulis G., de Graaf J., Couture P., Hegele R.A., Sniderman A.D. (2022). Physiological bases for the superiority of apolipoprotein B over low-density lipoprotein cholesterol and non-high-density lipoprotein cholesterol as a marker of cardiovascular risk. J. Am. Heart Assoc..

[43] Ghasempour G., Zamani-Garmsiri F., Shaikhnia F., Soleimani A.A., Hosseini Fard S.R., Leila J., Teimuri S., Parvaz N., Mohammadi P., Najafi M. (2024). Efficacy and Safety of Alirocumab and Evolocumab as Proprotein Convertase Subtilisin/Kexin Type 9 (PCSK9) Inhibitors in Familial Hypercholesterolemia: A Systematic Review and Meta-Analysis. Curr. Med. Chem..

[44] Vuorio A., Watts G.F., Schneider W.J., Tsimikas S., Kovanen P.T. (2020). Familial hypercholesterolemia and elevated lipoprotein(a): Double heritable risk and new therapeutic opportunities. J. Intern. Med..

[45] Dai H., Zhu Y., Chen Z., Yan R., Liu J., He Z., Zhang L., Zhang F., Yan S. (2023). Impact of alirocumab/evolocumab on lipoprotein (a) concentrations in patients with familial hypercholesterolaemia: A systematic review and meta-analysis of randomized controlled trials. Endokrynol. Polska.

[46] Blom D.J., Harada-Shiba M., Rubba P., Gaudet D., Kastelein J.J.P., Charng M.J., Pordy R., Donahue S., Ali S., Dong Y. (2020). Efficacy and safety of alirocumab in adults with homozygous familial hypercholesterolemia: The odyssey hofh trial. J. Am. Coll. Cardiol..

[47] Koren M.J., Sabatine M.S., Giugliano R.P., Langslet G., Wiviott S.D., Ruzza A., Ma Y., Hamer A.W., Wasserman S.M., Raal F.J. (2019). Long-term efficacy and safety of evolocumab in patients with hypercholesterolemia. J. Am. Coll. Cardiol..

[48] Roth E.M., Goldberg A.C., Catapano A.L., Torri A., Yancopoulos G.D., Stahl N., Brunet A., Lecorps G., Colhoun H.M. (2017). Antidrug antibodies in patients treated with alirocumab. N. Engl. J. Med..

[49] Buonuomo P.S., Mastrogiorgio G., Leone G., Rana I., Gonfiantini M.V., Macchiaiolo M., Vecchio D., Gnazzo M., Bartuli A. (2021). Evolocumab in the management of children <10 years of age affected by homozygous familial hypercholesterolemia. Atherosclerosis.

